# Can Patient Safety Incident Reports Be Used to Compare Hospital Safety? Results from a Quantitative Analysis of the English National Reporting and Learning System Data

**DOI:** 10.1371/journal.pone.0144107

**Published:** 2015-12-09

**Authors:** Ann-Marie Howell, Elaine M. Burns, George Bouras, Liam J. Donaldson, Thanos Athanasiou, Ara Darzi

**Affiliations:** 1 Department of Surgery and Cancer, Imperial College, St Mary’s Hospital, London, United Kingdom; 2 Institute of Global Health Innovation, Imperial College, St Mary’s Hospital, London, United Kingdom; University of Utah Health Sciences Center and ARUP Laboratories, UNITED STATES

## Abstract

**Background:**

The National Reporting and Learning System (NRLS) collects reports about patient safety incidents in England. Government regulators use NRLS data to assess the safety of hospitals. This study aims to examine whether annual hospital incident reporting rates can be used as a surrogate indicator of individual hospital safety. Secondly assesses which hospital characteristics are correlated with high incident reporting rates and whether a high reporting hospital is safer than those lower reporting hospitals. Finally, it assesses which health-care professionals report more incidents of patient harm, which report more near miss incidents and what hospital factors encourage reporting. These findings may suggest methods for increasing the utility of reporting systems.

**Methods:**

This study used a mix methods approach for assessing NRLS data. The data were investigated using Pareto analysis and regression models to establish which patients are most vulnerable to reported harm. Hospital factors were correlated with institutional reporting rates over one year to examine what factors influenced reporting. Staff survey findings regarding hospital safety culture were correlated with reported rates of incidents causing harm; no harm and death to understand what barriers influence error disclosure.

**Findings:**

5,879,954 incident reports were collected from acute hospitals over the decade. 70.3% of incidents produced no harm to the patient and 0.9% were judged by the reporter to have caused severe harm or death. Obstetrics and Gynaecology reported the most no harm events [OR 1.61(95%CI: 1.12 to 2.27), p<0.01] and pharmacy was the hospital location where most near-misses were captured [OR 3.03(95%CI: 2.04 to 4.55), p<0.01]. Clinicians were significantly more likely to report death than other staff [OR 3.04(95%CI: 2.43 to 3.80) p<0.01]. A higher ratio of clinicians to beds correlated with reduced rate of harm reported [RR = -1.78(95%Cl: -3.33 to -0.23), p = 0.03]. Litigation claims per bed were significantly negatively associated with incident reports. Patient satisfaction and mortality outcomes were not significantly associated with reporting rates. Staff survey responses revealed that keeping reports confidential, keeping staff informed about incidents and giving feedback on safety initiatives increased reporting rates [r = 0.26 (p<0.01), r = 0.17 (p = 0.04), r = 0.23 (p = 0.01), r = 0.20 (p = 0.02)].

**Conclusion:**

The NRLS is the largest patient safety reporting system in the world. This study did not demonstrate many hospital characteristics to significantly influence overall reporting rate. There were no association between size of hospital, number of staff, mortality outcomes or patient satisfaction outcomes and incident reporting rate. The study did show that hospitals where staff reported more incidents had reduced litigation claims and when clinician staffing is increased fewer incidents reporting patient harm are reported, whilst near misses remain the same. Certain specialties report more near misses than others, and doctors report more harm incidents than near misses. Staff survey results showed that open environments and reduced fear of punitive response increases incident reporting. We suggest that reporting rates should not be used to assess hospital safety. Different healthcare professionals focus on different types of safety incidents and focusing on these areas whilst creating a responsive, confidential learning environment will increase staff engagement with error disclosure.

## Introduction

Voluntary reporting of adverse events to external agencies was initiated in the industrial and transport sectors. In these sectors, good levels of operational safety are achieved within high-risk environments. [1] A database of patient safety incident reports (the National Reporting and Learning System; NRLS) was created within the National Health Service (NHS) in England in 2003. It is now the largest repository of such incidents in the world. Similar systems to capture adverse events have now been established in many other countries.[2] The NRLS was originally designed to enable analysis of frequently occurring and serious events. From these reports, the NRLS developed and issued national patient safety warnings and disseminated safety solutions to prevent such events recurring. Regulators currently scrutinize the rates of reported safety incidents to assess the relative safety of hospitals.[3]

High profile service failures within the NHS in the United Kingdom (UK) have raised public concern about preventable harm in healthcare and increased the demand for transparency and accountability. It is a reasonable expectation that the large volume of information collected through incident reporting should allow valid judgments about the risks to patients in one hospital compared to another. Indeed, a recent major enquiry into the safety failings in one English hospital expressed some incredulity that this was not already a routine component of monitoring of NHS performance.[3]

The main regulator of NHS hospitals, the Care Quality Commission, already assesses the rates of incident reporting for individual hospitals. The reporting rate reflects not only the true number of safety incidents within an organization but also the reporting behavior and culture within an institution. It is not clear whether examining NHS trust crude reporting rates distinguishes unsafe care or whether it merely reflects variation in reporting behavior. Hutchinson and colleagues examined NRLS data two years after incident reporting commenced in 2005 and, at that time, found no correlation between high reporting rates and poor hospital outcome. They concluded that the lack of such an association was almost certainly due to low reporting rates. [4] Since this study was published reporting rates have increased to over one million patient safety incidents a year, with significant variation between hospitals persisting.[5]

We set out the hypothesis that hospitals with better infrastructure, lower standardized mortality rates, higher patient satisfaction and less litigation would be better at reporting patient safety incidents. We aimed to establish what factors relate to the high reporting of no harm events and those incidents that lead to harm or even death. In addition, we aimed to assess what organizational culture factors correspond to a high reporting rate using a national staff survey.

## Methods

The study population included all reports of patient safety incidents from NHS acute hospital trusts during the period 1^st^ January 2003 to 31^st^ May 2013 obtained from the NRLS database. Although all NHS organizations are required to report incidents, those without inpatient provision, (e.g. Primary care and mental health services) were excluded from the present study. In addition, specialist hospitals such as neurosurgical or paediatric centers were excluded. These exclusions aimed to reduce the potential for bias and to ensure homogeneity of the analyzed hospital trusts. Primary care centers rarely report incidents and have different quality assessments. Specialist hospitals have different structural and process frameworks and therefore are not suitable for comparison.

### Data description

The NRLS was established by National Patient Safety Agency in 2003 and has accumulated over nine million reports since it began collection in 2003. All NHS staff are encouraged to report the patient safety incidents that they observe. A “patient safety incident” is defined as an event during an episode of patient care that had the potential to or did cause injury or harm to the patient. [6] Each report requires: demographic and administrative data: incident category, degree of harm, organization code, incident location, age, sex and ethnicity of patient and date and time of incident. The job description of the member of staff reporting is also captured. These are categorical variables, mainly captured in drop-down menus. There is also a free text section in which the reporter is asked to describe what happened and what action was taken as a result. This study examined the categorical data.

The reporter designates an incident’s severity as no harm, low harm, moderate harm, severe harm or death. Harm is defined as injury or complication leading to morbidity, mortality or increased length of stay. For the purposes of understanding what factors relate to reported harm versus no harm; low harm, moderate harm, severe harm and death were grouped as “harm”.

### Hospital characteristics

Several hospital characteristics were examined. (Table 1)

**Table 1:** Summary of datasets used to evaluate hospital factors influencing reporting rates.

**Hospital factor: Structure**	**Dataset**
Hospital size and number of critical care beds	Department of Health, Publications and statistics
Teaching Hospital Status	NRLS designation
Nurses and clinicians to beds ratio	The Information Centre
**Hospital factor: Outcome**	**Dataset**
Standardised Hospital Mortality Index	The Information Centre
Patient views on care	Care Quality Commission
Litigation Claims	National Health Service Litigation Authority
Litigation Payments	National Health Service Litigation Authority

Incident reporting rates for hospital trusts in the study population were obtained from the Organization Patient Safety Incident Reports. [7] Reporting rates were calculated per 100 admissions. The denominator of number of admissions was taken from the Hospital Episode Statistics.

The frequency of reported patient safety incident rates were correlated with hospital characteristics over the same time period. Factors associated with harm and deaths were examined in more detail. Hospital factors were considered to be either structural or performance-related.[8] No process factors were used, as there were not any nationally collected metrics that included data from all hospitals.

The hospital size was determined by two factors: the number of beds and staffing levels using routine NHS statistical sources. [9, 10] Total available bed numbers were calculated as the average daily number of open and staffed beds overnight. [10] The staffing rate was calculated as full time clinicians or nurses divided by available beds to allow comparison between hospitals trusts.

Summary Hospital-Level Mortality Indicator (SHMI) and Care Quality Commission (CQC), institutional level, patient survey data were used as outcome measures. SHMI figures were obtained from The NHS Information Centre [7] The indicator reports risk adjusted all-cause mortality 30 days post discharge and is derived from Hospital Episode Statistics/Office for National Statistics linked data as a risk-adjusted ratio of observed deaths over expected deaths. [11, 12] Risk-adjusted expected death rates are calculated for diagnostic groups taking into account admission method, age, gender and Charlson comorbidity Index.[13] During initial analysis there was significant association between SHMI and rate of reported death (relative risk = 0.02, 95%CI: 0.00 to 0.04,p = 0.03). (Table 2) As it was likely to be a confounder, it was removed from the analysis for factors influencing death rate.

**Table 2:** Correlations between different metrics of quality and safety.

Factor	Total reporting rate/100 admissions		“Death” rate/100 admissions	
	Spearman’s coefficient	P value	Spearman’s coefficient	P value
Beds/Hospital trust	-0.07	0.43	-0.04	0.66
Teaching Hospital status	0.10	0.25	0.07	0.40
Critical care beds	-0.05	0.57	-0.01	0.94
Clinicians/bed	-0.11	0.19	0.17	0.04
Nurses/bed	-0.01	0.87	0.14	0.09
SHMI	-0.01	0.91	0.16	0.05
Overall CQC score	0.09	0.30	0.05	0.54
NHS Litigation Authority claims	-0.16	0.10	0.06	0.55
NHS Litigation Authority claims/bed	-0.15	0.12	0.06	0.52
NHS Litigation Authority payments/bed	-0.12	0.25	0.08	0.45
NHS Litigation Authority payments	-0.16	0.11	0.07	0.51

The CQC is an independent regulator of health and adult social care in England. As part of its role it surveys patients’ experience of their hospital stay. Hospitals are ranked based on questions relating to overall quality of care. Data regarding claims and payments for litigation were obtained from the NHS Litigation Authority. [14]

### NHS staff survey

The NHS Staff Survey collects information from staff about working conditions in their NHS organization. The survey asks specific questions about adverse events and incident reporting. [15]

### Statistical analysis

Statistical analysis was performed with SPSS^®^ version 20.0 (SPSS, Chicago, Illinois USA.) For non-parametric variables median (interquartile range) values were given and non-parametric data were correlated using Spearman’s rank correlation coefficient. P<0.05 was considered significant for all tests.

Factors potentially influencing reporting frequency were evaluated using Pareto chart analysis to highlight the areas of vulnerability for patient safety. Pareto charts express categories in descending order of frequency using a bar graph whilst the cumulative total of values or occurrences for each category is represented by a line graph.

A logistic regression model was used to examine patient and staffing factors that relate to the level of harm as identified by the reporter. Separate linear regression models were created for modeling reporting rates and hospital factors harm versus no harm, and for death versus no death. Harm included low harm, moderate harm, severe harm or death, with no harm as the reference category. Factors with a significance level of <0.1 on bivariance analysis were included in the regression.

### Ethical approval

This research was approved by the Imperial College Joint Research Compliance Office Reference number 13SM0726. All patient records and information were anonymised and de-identified by the NRLS before being accessed by the research team, and therefore consent for use of data was not required

## Results

### Data quality analysis

A total of 148 hospital trusts reported to the NRLS over the ten-year study period. There were fifteen key variables that had greater than 80% complete data. (Table 3) Unfortunately variables such as patient ethnicity and member of staff reporting are not mandatory categories and are poorly completed. Overall reporting rates increased exponentially from 2003 to 2013. In total, 66, 931 incidents for acute hospitals were reported between 2003 and 2005 as the penetrance of the system increased. In 2013, during the one-year period, there were 1,093,091 reports.

**Table 3:** Data quality assessment: Incident demographic variables.

Variable type	Variable	%Complete
Incident variables	Unique incident identifier	100.00
	Date the report was exported to the NRLS cleansed	84.49
What happened	Free text description of what happened	99.38
	Incident category	99.39
	Degree of harm (severity)	99.39
Where incident occurred	Country	97.66
	NHS Organisation code	99.40
	Location of incident e.g. Ward	99.39
Patient demographics	Adult/Paediatric specialty	98.01
	Patient sex	80.73
	Age at time of incident	63.31
	Patient ethnic category	35.91
When incident occurred	Month and Year of Incident	91.74
	Date of incident	84.68
	Time	83.77
Who reported the incident	Staff type	16.06

### Trends in reported harm and death

**Fig 1:**
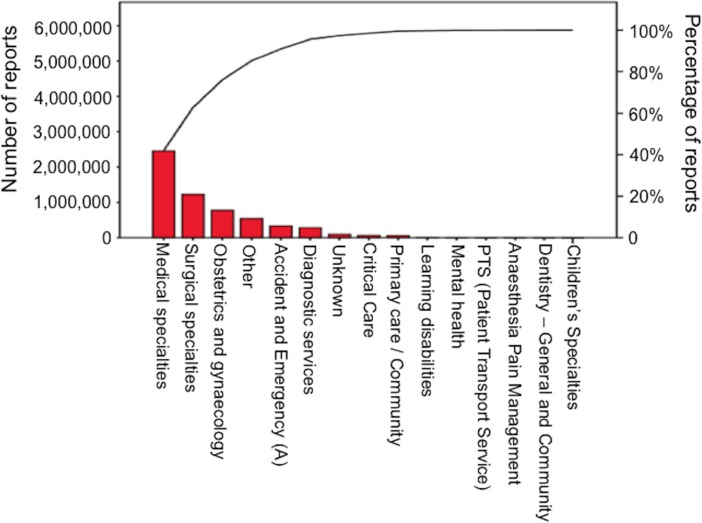
Pareto chart showing medial specialty and incident reporting frequency.

**Fig 2:**
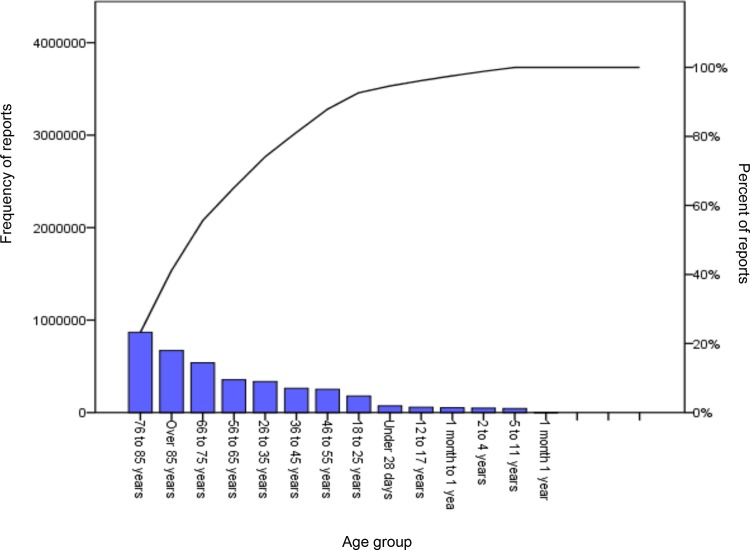
Pareto chart showing patient age group and incident reporting frequency.

Over 60% of reports concerned patients over the age of 65 years. The patients most likely to be included in an incident report were in the 76–85 year age group (867,548 reports) When harm was reported over half of such incidents involved patients in medical specialties. (Figs 1 and 2)

### Relationship between reported harm and patient or staff factors

Using binary multiple logistic regression analysis, the effects of patient age, gender, specialty, month, location, time and day of report on whether a harm report or no harm report were assessed.

The age group 76–85 years was most likely to have incidents reported [OR 1.49 (CI 1.44–1.54) p<0.05]. Male patients were less likely to have reported harm OR 0.93 (95%CI: 0.92–0.94), p<0.001. No harm incidents were most likely to be reported from pharmacy [OR 3.03 (95%CI: 2.04–4.55), p<0.001].

Patients were most likely to have a harm report during their stay if admitted under the medical specialties [OR 1.77 (95%CI: 1.71–1.83), p<0.001]. Obstetrics and gynaecology professionals reported the most near miss reports: OR 1.61 (95%CI: 1.12–2.27) (p = 0.009) Clinicians were slightly more likely to file a harm report than other specialties when adjusted for age, gender, month, location, time, weekend [OR 1.085 (95%CI: 1.050–1.121), p<0.01)].

### Relationship between reported death and patient and staff factors

Clinicians were significantly more likely to report deaths than other staff members [OR 3.04 (95%CI: 2.43–3.80) p<0.01]. When adjusted for all factors death was more likely to be reported at night [OR 1.25 (95%CI: 1.13–1.39), p<0.01].

### Relationship between reporting rates and hospital characteristics

Hospital level data were examined for 2011. There were 399,751 reports included during 2011. Of these reports, 11,5031 (28%) were harm reports and 688 (0.17%) were reported as deaths. The median number of reports across all hospital trusts was 5.87 [Inter-quartile Range (IQR) = 2.06] per 100 admissions and the median number of deaths reported was 0.01 (IQR = 0.01) per 100 admissions.

### Structural factors

The median number of full time clinicians per bed per hospital trust was 0.77 (IQR = 0.23). There were no significant associations between clinicians per bed and overall rate of reporting or reported deaths (Table 4). There was a significant negative association between clinicians per bed and rate of reported harm [Relative risk (RR) = -1.78, 95% Confidence Interval (CI): -3.33 to-0.23, p = 0.03] (Table 5).

**Table 4:** Associations between hospital characteristics and overall rate of reporting, multiple linear regression models.

Factor	B Coefficient	P value	Confidence intervals
Teaching Hospital status	0.74	0.16	-0.27 to 1.75
Clinicians/bed	-2.67	0.08	-5.66 to 0.33
Nurses/bed	0.81	0.32	-0.79 to 2.40
SHMI	-0.54	0.72	-3.54 to 2.45
Overall CQC score	0.03	0.27	-0.03 to 0.09
NHS Litigation Authority claims/bed	-5.00	0.49	-19.22 to 9.231

**Table 5:** Associations between hospital characteristics and rate of reported harm, multiple linear regression models.

Factor	B Coefficient	P value	Confidence intervals
Teaching Hospital status	0.12	0.65	-0.40 to 0.65
Clinicians/bed	-1.78	0.03	-3.33 to -0.23
Nurses/bed	0.82	0.05	-0.01 to 1.64
SHMI	-0.41	0.61	-2.00 to 1.15
Overall CQC score	0.02	0.14	-0.01 to 0.05
NHS Litigation Authority claims/bed	9.30	0.01	2.04 to 16.54

The median number of full time nurses per bed per hospital trust was 1.82 (IQR = 0.38). There were no significant associations between nurses per bed and overall rate of reporting or reported harm or deaths. (Tables 4 and 5)

Twenty-seven hospitals were classed as teaching hospitals and there were no significant associations with overall rate of reporting or reported harm or deaths. (Tables 4 and 5)

### Outcome factors

There were no significant associations between SHMI and overall rate of reporting or reported harm. (Tables 3 and 4)

The overall CQC survey response rate was 53%. [16] CQC median overall scores were 6.40 (IQR = 0.40). (Table 3) There were no significant associations between CQC scores and overall rate of reporting or reported harm or deaths. (Tables 4 and 5)

The median number of NHS Litigation Authority (LA) claims per hospital trust per bed was 0.06 (IQR = 0.00). There were no significant associations between claims and rate of reported harm or reported deaths. The number of claims negatively correlated with the reported harm rate (RR = 9.30 95%CI: 2.04 to 16.54, p = 0.01) (Table 5).

### Barriers to reporting: investigating relationship between reporting rates and NHS staff survey

The median number of staff per trust responding to the NHS Staff survey questions was 399 (IQR = 93.5). NHS staff survey questions related to incident reporting showed significant correlations between reporting rate and the following factors: hospital trusts that encourage reporting [r = 0.26 (p = 0.001)], keep reports confidential [r = 0.17 (p = 0.04)], keep staff informed about incidents [r = 0.23 (p = 0.01)] and feedback on changes made [r = 0.20 p = 0.02)]. Hospital trusts that penalized staff for incidents had a negative correlation with reporting rate [r = -0.18 (0.03)]. (Table 6)

**Table 6:** Correlations between NHS Staff survey questions on error reporting and reporting rate.

Question (summarized)	Total reporting rate/100 admissions	
	Spearman’s coefficient	P value
Have you seen any incidents in the last month?	0.09	0.29
Have you reported an incident in the last month?	0.18	0.03
Hospital trust treats staff involved in incidents fairly	0.07	0.37
Hospital trust encourages reporting	0.26	<0.01
Hospital trust treats reports confidentially	0.17	0.04
Hospital trust punishes people involved in incidents	-0.18	0.03
Hospital trusts takes action to prevent further incidents	0.13	0.11
Hospital trust informs staff about incidents occurring in the hospital trust	0.23	0.01
Staff get feedback about changes made as a result of reported incidents	0.20	0.02

## Discussion

The rate of patient safety incident reports in the NHS in England has increased exponentially since collection began. This reflects the emphasis placed on patient safety by successive governments and the willingness of front-line staff to provide information that is ultimately intended to reduce the risks of care.[17–19]

### Reporting trends

Our analysis reveals that patients most vulnerable to reported harm are elderly medical inpatients. This merely corresponds to the inpatient population as nearly two thirds of UK hospital admissions are patients aged over 65 years, and account for approximately 70% of bed days.[20] In addition to factors such as increased frailty over 85 year olds account for 25% of bed days and have, on average, a significantly longer hospital stay than younger patients.[20]

We observed that clinicians are significantly more likely to report a death than other members of staff, although there were lower rates of reports filed by clinicians overall, in keeping with other studies.[11, 21] Such variation may reflect the perceived level of responsibility for handling different levels of harm. Using reports of death to trigger safety initiatives may motivate clinicians better than near miss reports.[21]

Obstetrics and gynaecology patients were more likely to have a no harm event reported than any other specialty. This specialty has an established history of reporting and a strong safety culture supported through established national audits into all maternal and neonatal morbidity and mortality. This may account for the dedication to reporting all patient safety incidents.

The hospital setting in which patients were most likely to have a near miss reported was in pharmacies. This excellent level of reporting may be explained through the process of medicine reconciliation. This is where medication errors are scrutinized closely and is a National Institute for Health and Clinical Excellence (NICE) guideline and shown to be effective in preventing harm.[22] The alternate explanation is that more near misses occur in pharmacy.

### Total reporting rate and hospital characteristics

Hospitals with better reporting records do not have particular differentiating hospital characteristics. (Tables 2 and 4) Staffing levels and teaching hospital status did not impact significantly on reporting rates. Unlike other studies we were unable to find an association between nursing staff levels and reported events. Our study took into account other organizational factors in the analysis.[23, 24]

The SHMI for hospital trusts had no demonstrable relationship with overall reporting rates. It was hypothesized that a low SHMI would correlate with a high overall reporting rate, suggesting that a hospital with a lower unexpected mortality rate would have a stronger safety culture, reporting and learning from incidents more frequently and therefore tackling failures that lead to patient harm. This was not the case. (Table 4) There were no associations between overall reporting rates and patient satisfaction and care as measured through the CQC survey or as stated hospital trust SHMI. Litigation payments were not correlated with overall reporting rates, but there was a significant negative association with claims and reporting rates.

It is important to note that there were no significant relationships between overall reporting rates and most hospital structure and outcome factors. This is in accordance with earlier studies and persists despite of increased reporting rates.[25] When Sari and colleagues, in 2007, examined adverse events recorded in case notes they found that the NRLS identifies only 5% of errors that cause harm to patients.[26] The NRLS cannot in its current form quantify the safety of a hospital. It is important that the data are not used to draw conclusions that are clouded by non-responder bias.

This problem is not unique to the NHS system. A report in 2012 from the inspector General of the Department of Health and Human Services in the United States found that only 14% of adverse events experienced by Medicare patients are captured.[27] The report noted that incident reporting systems were relied on to identify safety problems, although administrators were aware the data were incomplete. Reasons for low reporting included staff not being aware of what events constituted harm.[27] This report recommended that the Agency for Healthcare Research and Quality (AHRQ) create a list of reportable events in collaboration with the Centers for Medicare and Medicaid Services.

### Reported harm rate and hospital characteristics

There were some associations found when incidents that caused patient harm were separated out and correlated with hospital characteristics.

Clinician-bed ratio corresponded to a significant reduction in the rate of harm reported, (although not having a relationship with overall reporting rates that included near misses). (Table 5) It may be that care received by patients is safer when clinician staffing is increased. Similarly, a study by Ghaferi et al also found that reduced rates of failure to rescue (death after a treatable surgical complication) in England were associated with a higher number of doctors per bed.[28]

### Reporting rates and litigation claims

There was a positive association between litigation claims and reported harm, when analysed separately from overall reporting rates. (Table 5) This is an interesting finding that contrasts to the negative association between litigation claims and overall reporting rates. One interpretation may be that hospitals reporting more have a stronger safety culture and therefore are less likely to have patients claiming for malpractice.

With respect to the positive association between claims and harm specific reports we suggest that generally staff may report specific incidents of harm when they are aware of the potential for patient claims and this may be to mitigate the litigation or provide real time documentation and accountability. This may be an area for further study if hospitals can use specific harm reporting data to identify areas of potential litigation risk.

### Insights from the NHS Staff Survey

Previous studies found relationships between high reporting rates and safety culture as assessed by the NHS Staff Survey. [25] Potential reasons for health care workers underreporting include concerns over reputation or peer disapproval, lack of meaningful feedback from the system or uncertainty regarding who was responsible for reporting.[11, 12, 29] The NHS Staff survey questions reflected NRLS reporting rates because hospital trusts where people stated that they reported events, the reporting rate was higher. Hospital trusts that encouraged reporting, had imposed confidentiality on reports, fed back to staff about incidents and promoted change had significantly higher reports. Hospital trusts that were deemed to have punished reporters had significantly lower reporting rates.

### Suggestions to improve learning from reporting

There is potential for redeveloping the data collection process to facilitate specialty based reporting, a method that has been successfully pioneered by the Australian Incident Monitoring System. [30] Accurate measurement of incidents requires standardized definitions of types of adverse event or complications for the given specialty and a minimum dataset is crucial for data homogeneity. [31] Our suggestions are two-fold. The first is to more tightly define a few specific incidents to be reported nationally so that staff can focus their reporting efforts. This has been adopted successfully in other systems, such as the Hong Kong system where specific patient safety incidents including never events are focused on and mandated. Secondly we suspect the true value of the NRLS reports lie in the patterns and trends picked up in the detail of the "free-text" section of the incidents. It may be more useful to focus local reporting systems on trying to capture why incidents occur rather than how often they occur. The current database should be fully exploited to understand the system failures that lead to patient harm. Methods for rapid free text analysis must be developed to enable real-time warning systems for at risk specialties or institutions. [32]

### Limitations

This study was limited by the paucity of commonly used national measures of hospital quality available to compare with reporting rates. These measures are often proxy metrics for quality and safety and all of them are prone to criticism.

Staffing and bed numbers are basic methods for understanding hospital structure and do not fully assess the complexities of hospitals as organizations. Despite this staffing numbers per bed have been reported to relate to patient safety and outcome, increased nurse staffing has been associated with lower adverse events and reduced hospital related mortality. This may justify our use of these measures for comparison and assessment.[2, 33–38] Future studies should include other structural factors such as the availability of appropriate IT equipment to facilitate reporting to fully understand discrepancies in reporting rates between hospitals.

The outcome measures we used to compare with incident rates are well known methods for assessing and benchmarking hospital performance.

SHMI is a commonly used measure of hospital performance and measures deaths adjusted for comorbidities. There has been criticism in the literature of SHMI as an outcome measure. The indicator relies on routinely collected data that can be inaccurate. A recent study showed that there were only weak associations between the proportion of avoidable deaths and the SHMI and that there were few significant differences between hospitals when avoidability of death was assessed. [39] However excess mortality as an endpoint is a useful broad indicator of quality and the SHMI has been recognized as transparent and reproducible. It is currently used as an acceptable method to flag hospitals that may be poorly performing.[40] Therefore we suggest that it was an appropriate standard to correlate with reporting rates. Future studies should seek to compare hospital reported death figures with hospital avoidable mortality demonstrated using retrospective case note review.

CQC patient survey results are outcome factors commonly used to characterize institutions.[35, 41, 42] With respect to using CQC patient survey results there is potential for bias in relying on the patient’s perception of care. Patients may over-estimate safety within an institution as they are not necessarily aware of safety issues. [43] However we wanted CQC survey results to be included as it is another dimension in the established definition of hospital quality.[44] The role of patients in monitoring their own care is becoming increasingly important.

The factors used in this study attempted to encapsulate measurable features of hospital care. This study chose reproducible, routinely collected data representing quantitative factors relating to hospital care, but the authors acknowledge that some organizations may have process factors, improved teamwork, communications and safety strategies, which have not been assessed in this study and that may well impact on reporting rates of patient safety incidents.[45, 46] This does limit the full assessment of whether reporting rates relate to hospital quality of care.

Using the staff survey has limitations with respect to assessing the quality of a hospital trust, as was shown by Pinder et al where there were weak negative correlations of staff survey results with mortality statistics.[47] However the survey does correlate with patient experience and takes views from a wide spectrum of thousands of staff members. We used the staff survey to understand what views regarding reporting influenced reporting rates and these were consistent in suggesting that staff perception is a strong moderator of reporting rate.

### What this study adds

It has been suggested that voluntary reporting may not be the best way to gather accurate information regarding how safe a hospital is.[48, 49] Particularly as public attention has been drawn towards patient safety and incident reporting, the variation in rates must be explained adequately.[50] Our findings agree that reported rates of events do not correlate strongly with other measures of hospital structure or performance. We suggest that to understand how safe a hospital is other data sources must be used. Using reporting rates, as an indicator of the relative safety or quality of a hospital trust is likely to be inaccurate. [51] When reports are separated into incidents that cause harm versus no harm there does seem to be a relationship between harm levels and clinician staffing. There is also correlation between harm and litigation claims. Looking at reported harm rates may be a useful area for further study. This study also shows that different specialties and staff focus on reporting different levels of patient harm. There was correlation between high reporting rates and confidential, but open reporting environments. Tailoring reporting to individual staff concerns may create better engagement from frontline staff.

## Conclusions

The NRLS is the largest patient safety reporting system in the world. This study did not find many hospital characteristics that significantly influenced overall incident reporting rates. There were no relationships between size of hospital, numbers of staff, mortality outcomes or patient satisfaction outcomes and reporting rates. The study did show that hospitals where staff reported more incidents had reduced litigation claims and when clinician staffing is increased fewer incidents reporting patient harm are reported, whilst near misses remain the same. Certain specialties report more near misses than others, and doctors report more harm incidents than near misses. Staff survey results showed that open environments and reduced fear of punitive response increases reporting. We suggest that reporting rates should not be used to assess hospital safety. Different healthcare professionals focus on different types of safety incident and focusing on these areas whilst creating a responsive, confidential learning environment will increase staff engagement with error disclosure.
